# Medication Management in Patients With Polypharmacy in Primary Care: A Scoping Review of Clinical Practice Guidelines

**DOI:** 10.1111/jebm.70015

**Published:** 2025-03-20

**Authors:** Loes Engels, Marjan van den Akker, Petra Denig, Henri Stoffers, Heike Gerger, Jolijn Bohnen, Jesse Jansen

**Affiliations:** ^1^ Department of Family Medicine Care and Public Health Research Institute Maastricht University Maastricht the Netherlands; ^2^ Institute of General Practice Goethe University Frankfurt Frankfurt am Main Germany; ^3^ Academic Centre of General Practice Department of Public Health and Primary Care Katholieke Universiteit Leuven Leuven Belgium; ^4^ Department of Clinical Pharmacy and Pharmacology University Medical Center Groningen University of Groningen Groningen the Netherlands; ^5^ Department of Clinical Psychology Faculty of Psychology Open University of the Netherlands Heerlen the Netherlands

**Keywords:** medication therapy management, patient participation, polypharmacy, practice guideline, shared decision‐making

## Abstract

**Objective:**

Inappropriate polypharmacy increases the risk of medication‐related issues. Adequate management of polypharmacy is a challenge involving different healthcare professionals, complex decision‐making and ideally including patient involvement. The objective of this scoping review was to provide an overview of national recommendations for medication management of patients with polypharmacy in primary care.

**Methods:**

A scoping review of clinical practice guidelines focusing on medication management in adults with polypharmacy, applicable to primary care was performed. Databases (G‐I‐N, Turning Research into Practice and PubMed), network, and a global report were screened for guidelines published after 2000 in English, Dutch, German, Spanish, French, or Russian. Raw data were extracted in duplicate using an extraction framework focusing on strategies, involvement of professionals, patient involvement, and implementation. Qualitative content analysis was used. Guideline quality was assessed using AGREE‐II. The study was registered with the Open Science Framework.

**Results:**

Eight guidelines originating from eight countries were included. The most common recommended strategy was a medication review conducted by a general practitioner and/or a community pharmacist. Tasks and target population differed per guideline. Most guidelines recommended involving the patient in the process, mostly to elicit the patient's experiences and treatment goals. Few guidelines included advice on the implementation of recommendations. Three out of eight guidelines were of good quality (AGREE‐II score >70% in 5/6 domains).

**Conclusions:**

Most guidelines recommended a medication review, with patient involvement, as a strategy for medication management in polypharmacy in primary care. Guidance on task division and implementation of guidelines in practice was less clear. This review illustrates room for guideline improvements.

## 1 Introduction

The approach to polypharmacy is an important topic [1]. Appropriate polypharmacy (i.e., rational prescribing of multiple medications) can be therapeutically beneficial. However, polypharmacy may also include inappropriate prescribing of medication, which has been shown to increase the risks of (potentially avoidable) medication‐related problems [1, 2, 3, 4, 5]. Examples include non‐adherence, adverse drug reactions (ADRs), hospital admissions, and mortality [4, 6, 7].

Different strategies for the adequate management of polypharmacy had been described in the literature and in clinical practice guidelines, further referred to as “guidelines” [5, 8, 9]. In a systematic review of guidelines on the management of patients with multimorbidity and polypharmacy, conducting medication reviews was identified as a key strategy to address (inappropriate) polypharmacy [10]. A medication review is a structured evaluation of a patient's medication to optimize medication use and improve health outcomes [11]. Performing a medication review seems to be an efficient way to reduce inappropriate polypharmacy and improve medication‐related outcomes [12, 13, 14].

Patients with polypharmacy often utilize multiple healthcare services, leading to potential challenges in coordinating medication management. These can be attributed to the involvement of various prescribers and inadequately defined roles and responsibilities [2, 6, 15]. Proper management of polypharmacy requires the combined knowledge of the different healthcare professionals involved and includes complex decision‐making with patient involvement [5].

In many healthcare systems, the management of polypharmacy is conducted in primary care, where the general practitioner (GP) or the community pharmacist has an overview of the medication used by patients. When referring to community pharmacists, this may also include other pharmacists working in primary care, such as practice‐based pharmacists. Teamwork is recommended for medication reviews but workload pressure may limit the contribution of, for example, the GP [16]. Community‐pharmacist‐led medication reviews can positively impact healthcare utilization and clinical outcomes [17]. However, it is unclear whether explicit recommendations have been made on the roles of different healthcare professionals in medication management.

Taking patient preferences into account is considered another vital element of medication reviews. It should improve patients’ quality of life and reduce the number of health problems that impact their daily lives [18]. Although eliciting patient preferences and exploring perceived medication‐related problems is considered relevant for adequate medication management [1, 2], it is unclear to what extent guidelines recommend the involvement of patients in a shared decision‐making process.

Healthcare contexts vary from country to country and recommendations for polypharmacy management may differ depending on the type of organization developing the guideline. Therefore, this scoping review aimed to provide an overview of national recommendations for medication management of patients with polypharmacy in primary care. We aimed to identify recommendations for different medication review strategies, the roles of different healthcare professionals—particularly GPs—the inclusion of patient perspectives in the medication management process, and strategies for implementing these recommendations in clinical practice.

## 2 Methods

We conducted a scoping review to identify recommendations for medication management strategies in patients with polypharmacy [19]. The PRISMA guidelines for scoping reviews (PRISMA‐ScR) were followed to ensure adequate reporting [20]. We registered the priori protocol with the Open Science Framework [21].

### 2.1 Eligibility Criteria

We included guidelines with recommendations applicable to primary care for medication management or medication review in polypharmacy among adults. We defined guidelines as: *“Statements that include recommendations intended to optimize patient care. They are informed by a systematic review of evidence and an assessment of the benefit and harms of alternative care options”* [22]. Inclusion and exclusion criteria are shown in Table S1.

### 2.2 Search Strategy

The search strategy included three steps. To identify grey literature, we first identified available guidelines from the WHO report “Medication Safety in Polypharmacy” [1]. In addition, relevant international networks in which members of the team participated were consulted by email to identify national guidelines from as many different countries as possible. Finally, a search strategy for searching electronic databases was developed based on terms used in available guidelines and index terms (Supplementary Material A). The following databases were searched: two specific guideline databases (Guidelines International Network [23], Turning Research into Practice [24]) and PubMed [25]. The initial timeframe for undertaking this scoping review was from November 1, 2022 until July 31, 2023. The search was repeated in April 2024, when no additional guidelines were identified. The reference lists of included records were screened for any additional eligible guidelines. One researcher (LE) performed an electronic search in all databases.

### 2.3 Guideline Selection

All identified records were collected and uploaded to EndNote 20.3, 2022 (Clarivate Analytics, PA, USA). Duplicates were removed. Two researchers (LE and HG) independently performed the screening. First, all records were screened for potentially relevant guidelines using the information provided in titles and abstracts. Irrelevant records were excluded. Next, both researchers independently assessed the full texts of all potentially relevant guidelines against the inclusion and exclusion criteria (Table S1). Any disagreements between both researchers were resolved through discussion within the research team.

### 2.4 Data Extraction

A priori data extraction framework was developed (LE) and was tested by the research team (LE, JJ, HS, MvA, PD). The framework was updated throughout the data extraction process (Supplementary Material B). Key elements for data extraction were based on research questions and the exploration of a few polypharmacy guidelines. They included recommendations for medication management strategy, patient involvement therein and the underlying framework, healthcare professionals’ roles in the process, and the implementation of the recommendations in practice. Raw data extraction was performed in duplicate (LE and JJ, MvA, or PD). General characteristics of each guideline were extracted, including author name(s), year of publication, country of publication, target setting, target population, definition of polypharmacy used in the guideline, aim of the guideline, and stakeholders involved in the development of the guideline.

### 2.5 Data Analysis

One researcher (LE) performed a descriptive qualitative content analysis of the recommendations extracted from the guidelines, resulting in coding and mapping key elements within the a priori defined data framework [26].

### 2.6 Quality Appraisal

We assessed the quality of all included guidelines using the Appraisal of Guidelines Research and Evaluation II (AGREE‐II) instrument [27]. The AGREE‐II consists of 23 items distributed across six domains (scope and purpose, stakeholder involvement, rigor of development, clarity and presentation, applicability, and editorial independence). Two researchers (LE and FU) independently appraised each guideline. The AGREE‐II instructions were followed to calculate domain scores. Domain scores ≥ 70% are considered adequate [27].

## 3 Results

### 3.1 Guideline Selection

**FIGURE 1:**
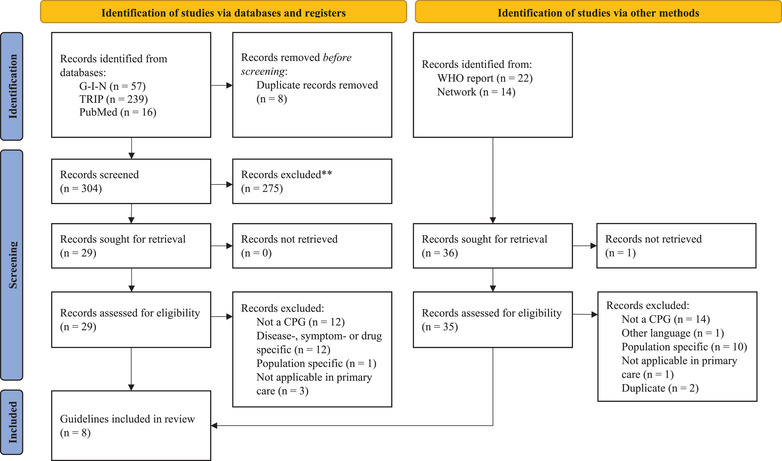
Flowchart of search strategy and clinical practice guideline selection. CPG, clinical practice guideline; G‐I‐N, Guidelines International Network; TRIP, Turning Research into Practice; WHO, World Health Organization.

A total of 348 records were identified, and 340 remained after removal of duplicates. Eight guidelines met the inclusion criteria (Figure 1).

### 3.2 General Characteristics

The general characteristics of the eight included guidelines are presented in Table 1. The definitions of polypharmacy varied. Older adults were the target population for most guidelines. Other target populations were patients with polypharmacy or frailty. The length of the guidelines ranged from 14 to 199 pages.

**TABLE 1:** General characteristics of included clinical practice guidelines.

Country of origin	Year	Guideline title [original]	Author(s)	Aim	Target setting	Target patient population^*^	Definition polypharmacy	Length (pages)
The Netherlands	2012 + 2019	Multidisciplinary Guideline Polypharmacy in the Elderly [Multidisciplinaire Richtlijn Polyfarmacie bij ouderen] [28] + Medication Review Module [Module Medicatiebeoordeling] [29]	Dutch College of General Practitioners Netherlands Society for Clinical Geriatrics Order of Medical Specialists	Effective pharmacotherapeutic care and multidisciplinary management of polypharmacy (guideline) + Adjustment of the methodology of medication assessment to apply it optimally in practice (module)	Primary and secondary care	Older patients with high risk of harm by polypharmacy (guideline) + Patients with polypharmacy aged 70 or older (module)	The chronic use of five or more different (ATC3 level) medications by one patient	199 (guideline) + 26 (module)
Mexico	2013	Clinical Practice Guideline Pharmacological Prescription in the Elderly [Guía de práctica clínica Prescripción farmacológica en el adulto mayor] [30]	Mexican Social Security Institute	Improve the quality of prescribing, through prevention and detection of inappropriate medication to reduce ADRs, deterioration of health and unjustified spending of resources	Primary and secondary care	Older adults	Simultaneous use of four or more medications	14
Wales	2014	Polypharmacy: Guidance for Prescribing [31]	All Wales Medicines Strategy Group	To address problems associated with the management of polypharmacy	Primary care	Patients with polypharmacy, indications of shortened life expectancy, and frail and older people	The use of at least four or five medications (distinction between appropriate and problematic)	34
United Kingdom	2015	Medicines Optimisation: The Safe and Effective Use of Medicines to Enable the Best Possible Outcomes [32]	National Institute for Health and Care Excellence	Safe and effective use of medication in health and social care for people taking 1 or more medications. To ensure that medication provide the greatest possible benefit to people	Primary care	People taking 1 or more medications	The use of multiple medications (distinction made between appropriate and inappropriate)	41 (guideline) 220 (full version)
Japan	2018	Guidance on Appropriate Medication for Elderly Patients [33]	Ministry of Health, Labor and Welfare	Optimize medication therapies for geriatric patients (avoidance of ADEs and inadequate medical care, improvement of medication adherence)	Primary care	Older people aged 65 or older (particular focus on 75 and over)	Polypharmacy denotes not simply using numerous medications concurrently, but rather the various concerns to which this can lead (risk of ADEs, medication errors, decreased adherence)	39
Scotland	2018	Polypharmacy Guidance Realistic Prescribing, 3rd Edition [34]	Scottish Government Polypharmacy Model of Care Group	To provide resources, expertise and insight for all involved with polypharmacy management	Primary and secondary care	Those with the greatest frailty, using the most medications and taking high‐risk medications	The use of two or more medications	83
Australia	2020	Guidelines for Comprehensive Medication Management Reviews [35]	Pharmaceutical Society of Australia	To identify, resolve and prevent medication‐related problems, and optimize medication use in partnership with medical practitioners and patients	Community pharmacists (primary and secondary care)	Not stated	Not stated	24
Germany	2021	General Practitioner Guideline on Polypharmacy. Recommendations for Dealing with Polypharmacy in Adults and Geriatric Patients. [Hausärztliche Leitlinie Multimedikation. Empfehlungen zum Umgang mit Multimedikation bei Erwachsenen und geriatrischen Patienten] [36]	German Society for General Medicine and Family Medicine	To assist GP with systematically evaluating medication therapy within the framework of the prescription decision. To contribute to recognizing, avoiding, or correcting the problems of over‐, under‐ and incorrect treatment that occur with polypharmacy	Primary care	All patients with polypharmacy and multimorbidity, excluding patients with palliative treatment	The prescription of five or more medications	155

Abbreviations: ATC, anatomic therapeutic classification; ATC3 level, equal therapeutic subgroups in the ATC; ADRs, adverse drug reactions, ADEs, adverse drug events; GP, general practitioner.

* The target patient population of the guideline may differ from the target patient population for conducting a clinical medication review. Not all guidelines focus on a population with polypharmacy per se, but in order to be eligible for the review, a significant proportion had to refer to polypharmacy.

### 3.3 Overview of Medication Management Recommendations

Table 2 shows an overview of different categories of recommendations extracted from the guidelines. All but one guideline (Mexico) recommended a medication review as a strategy for medication management. Three guidelines (The Netherlands, Mexico, Germany) additionally recommended a type of warning system in medication management. Examples of these warning systems are the Screening Tool of Older Persons’ Potentially Inappropriate Prescriptions (STOPP)—Screening Tool to Alert doctors to Right Treatment (START) or the Beers criteria to detect potentially inappropriate prescriptions in the elderly, or for instance local warning systems in electronic patient files to give an alert for patients that are up for a medication review. Medication reconciliation was recommended in one guideline (United Kingdom). The healthcare professionals most recommended to be involved in medication management were GPs and community pharmacists, specialists and (practice) nurses. Pharmacy assistants (The Netherlands) or dentists (Japan) were each mentioned in one guideline. Shared decision‐making, communication and patient education were the most common approaches recommended for patient involvement. Three (The Netherlands, Germany, United Kingdom) out of eight guidelines provided some advice on implementing recommendations in practice.

**TABLE 2:** Overview of categories of recommendations per clinical practice guideline (*n* = 8).

Content of guideline recommendations		The Netherlands	Mexico	Japan	Germany	United Kingdom	Wales	Scotland	Australia
Medication management	Medication review	•		•	•	•	•	•	•
	Warning system to detect inappropriate prescription	•	•		•				
	Risk assessment of the need for further evaluation of medication use	•							•
	Comprehensive geriatric assessment		•	•					
	Practical solutions (e.g., to identify patients that meet the criteria)	•			•				
	Medicines reconciliation					•			
Healthcare professionals involved	General practitioner	•	•	•	•	•	•	•	•
	Community pharmacist	•		•	•	•	•	•	•
	(Practice) nurse	•		•				•	•
	Medical specialist	•	•			•			•
	Multidisciplinary approach	•				•			•
	Pharmacy employee	•							
	Dentists			•					
Patient involvement	Communication	•			•	•	•	•	•
	Shared decision‐making				•	•	•	•	•
	Patient education			•			•	•	•
	Patient decision aid					•		•	
	Patient‐centered care			•					•
Implementation in practice	Implementation plan	•				•			
	Practice routine for conducting medication management				•				
	Data and technology for implementation					•			

### 3.4 Medication Reviews

The target population, definition, aim, and elements of a medication review as reported in the included guidelines are shown in Table 3. The target population differed largely among guidelines; only a few used explicit criteria. The most common elements to select the target population, described in six out of eight guidelines, were older age, number of medication used, and high‐risk medication or frailty. The definition or the aim of medication reviews was not mentioned in all guidelines. When mentioned, “structural evaluation” was commonly used to describe medication reviews. Although the specific elements of a medication review varied between guidelines, they all included gathering patient‐information, communicating with the patient, developing a management plan, and monitoring medication changes. Two guidelines (United Kingdom, Wales) specified different levels of conducting a medication review, such as a prescription review or a concordance and compliance review, in addition to a full clinical face‐to‐face medication review. Unique elements were the recommendations for a geriatric assessment in the Japanese guideline, an explicit consultation between community pharmacist and GP in the Dutch guideline, the explicit attention for dose reduction of medication in the Japanese and Welsh guidelines and the assessment of treatment burden in the German guideline.

**TABLE 3:** Target population, definition, aim, and elements of medication reviews as recommended per guideline (*n* = 7 out of 8 guidelines).

Guideline	Medication review			
	Target population	Definition	Aim	Elements
The Netherlands	Patients > 75 years of age and with chronic use of ≥ 10 medications and/or established frailty	An integrated assessment of the pharmacotherapy by the patient (or caregiver), doctor and pharmacist based on a structured, critical evaluation of the medical, pharmaceutical and usage information with the aim of optimizing the effectiveness of the pharmacotherapy and reducing of the risk of pharmacotherapeutic problems	To check whether current medication use is appropriate taking into account the patient's wishes and needs, professional standards/guidelines, the patient's diagnosis; has an appropriate balance of efficacy and safety, and is as desired by the patient.	Systematic Tool to Reduce Inappropriate Prescribing: Pharmacotherapeutic Anamnesis of current drug use, use‐related problems, experiences, concerns, expectations and beliefs with regard to medication and personal treatment goals Pharmacotherapeutic Analysis of potential pharmacotherapeutic problems using explicit criteria Consultation between pharmacist and GP (preferably verbally): draw up pharmacotherapeutic treatment plan and agree on who communicates with the patient and implements Feedback to patient: pharmacotherapeutic treatment plan is discussed with patient and, if necessary, adjusted
Japan	Not specified	Not stated	Necessary for the patients with problems related to polypharmacy based on a result of a comprehensive geriatric assessment	General rules of prescription review: Comprehensive Geriatric Assessment including an assessment of cognitive function, activities of daily living, living environment, and drug preference Monitoring physiological functions Prescription priority and dose reduction/drug discontinuation. Take into account the number of medications, the number of tablets, frequency of dosing, combination medication, and improving adherence
Germany	Patients with multiple medications (≥ 5 long‐term use drugs) and multimorbidity (≥ 3 chronic diseases)	Not stated	Not stated	Information collection: preexisting conditions, current complaints, clinical status and current relevant laboratory values, prescriptions/self‐medication, information on lifestyle factors, psychosocial context, and therapy goals of the patient Assess treatment burden Evaluate medication in a structured manner The medication process: inventory and evaluation, coordination with the patient, regulation proposal and communication, drug delivery, drug use/self‐management, monitoring/follow‐up
United Kingdom	Adults, children and young people with polypharmacy Adults, children and young people with chronic or long‐term conditions Older people	A structured, critical examination of a person's medication with the objective of reaching an agreement with the person about treatment, optimizing the impact of medication, minimizing the number of medication‐related problems and reducing wasteDifferent types of medication review: Level 0—unstructured review Level 1—prescription review Level 2—concordance and compliance review Level 3—clinical medication review	Not stated	During a structured medication review, take into account: the patient's views and understanding about their medication the patient's concerns, questions or problems with the medication all prescribed, over‐the‐counter and complementary medication medication safety, function, appropriateness the patient's risk factors for developing adverse drug reactions any monitoring that is needed
Wales	Patients with polypharmacy Patients with indications of shortened life expectancy Frail and elderly patients	A structured, critical examination of a patient's medication with the objective of reaching an agreement with the patient about treatment, optimizing the impact of medication, minimizing the number of medication‐related problems and reducing waste A medication review can be described in differing levels dependent on the depth of the review from Level 0 (unstructured, opportunistic) to Level 3 (full clinical face‐to‐face review of medication and condition)	Not stated	GPs are required to conduct medication review on an annual basis: Evidence‐based guideline/consensus for the medication; for the indication; at the current dosage; in this patient's age group Risks versus benefits analysis Dose, formulation and dosing Monitoring Hospital admission or changes Vital hormone replacement Rapid symptomatic deterioration prevention Day to day benefit Dose reduction without risk Conditions still relevant Either continue, reduce dose, and monitor or consider stopping the medication in conjunction with patient
Scotland	Patients: Aged 50 years and older and resident in a care home Approaching the end of their lives. Prescribed 10 or more medications On high‐risk medication (as defined by the Case Finding indicators)	7‐step structure for both the initiation of new and the review of existing treatments	To address all six dimensions of quality in medication management: efficacy, safety, efficiency, timely, equity, and acceptability	Step 1: (Aim) What matters to the patient? Identify aims and objectives of drug therapy Explain any key information Establish treatment objectives with the patient Step 2: (Need) Identify essential drug therapy List of medication Ensure the patient understands the importance Step 3: (Need) Does the patient take unnecessary drug therapy? Verify medication function in the therapeutic goals or outcomes Review preventative treatment Lifestyle changes Step 4: (Effectiveness) Are therapeutic objectives being achieved? Check if treatment choice is the most effective Patient nonadherence should be investigated Dose titration Step 5: (Safety) Is the patient at risk of ADRs or suffers actual ADRs? Identify the presence of ADRs Step 6: (Efficiency) Is drug therapy cost‐effective? Opportunities for cost minimization should be explored Ensure prescribing is in line with current formulary recommendations Step 7: (Patient‐centered) Is the patient willing and able to take drug therapy as intended? Does the patient understand the outcome Ensure drug therapy is tailored to patient preferences Agree and communicate plan with patient
Australia	Based on the individual's clinical status	A multidisciplinary activity whereby the risks and benefits of each medication are considered with the patient (and/or their carer, representative or substitute decision‐maker) and decisions are made about their future medication regime	To improve the appropriateness of medication, reduce harm and improve health outcomes, while incorporating the patient's preferences, beliefs, attitudes, and priorities	Collation and consideration of information from referral and from patient consultation, discussion with carers, review of clinical notes and pathology results, and review of information provided by the pharmacy Identification of medication‐related problems and development of recommendations to address problems. Discuss and clarify potential problems with referring medical practitioner Preparation of comprehensive medication management review report that incorporates findings and recommendations to address patient's medication‐related problems and provide to medical practitioner. Review report should indicate follow‐up Medical practitioner assesses report and, in consultation with patient, prepares Medication Management Plan

Abbreviations: ADR, adverse drug reaction; GP, general practitioner.

### 3.5 Healthcare Professionals

The GP was recommended to play a vital role in medication management in all guidelines (Table 4). The community pharmacist was recommended to be involved in medication management in all guidelines except one (Mexico). In six guidelines, the roles of the GP and the community pharmacist partly overlapped. For example, conducting the medication review, assessing risk and clinical need, and informing the patient were roles that could be performed by either the GP or the community pharmacist. One guideline (Australia) specifically recommended that the community pharmacist conducts the medication review, after which the GP can develop a management plan. In two guidelines (Japan, Germany), the community pharmacist was given specific tasks concerning the provision of information about the medication to support the GP or the patient.

**TABLE 4:** Roles of the general practitioner and pharmacist in medication management described per guideline (*n* = 8 out of 8 guidelines).

Guideline	Healthcare professional	
	GP	Community pharmacist
The Netherlands	Ensure patients are aware of information about medication assessment Provide short questionnaires or leaflets Make risk assessment and determine further evaluation of medication use in patient Interview patient, discuss management plan and follow‐up Management plan is drawn up and evaluated under joint responsibility of doctor and pharmacist	Ensure patients are aware of information about medication assessment Provide short questionnaires or leaflets Make risk assessment and determine further evaluation of medication use in patient Interview patient, discuss management plan, and follow up Management plan is drawn up and evaluated under joint responsibility of doctor and pharmacist
Mexico	No specific roles identified	Not included in recommendations
Japan	Central role in medication therapy Collaboration to confirm rationales behind patient's prescriptions Take care of patient after hospital discharge for continuous prescription review	Central role in medication therapy Centralize information, make prescription appropriate, and cooperate with patient's primary care physician Provide information on medication prescription to healthcare professionals involved in patient's care
Germany	Agree about collaboration and form of contact with pharmacist Annual meeting of GPs and pharmacists to clarify current problems in prescription of medication and delivery Organize collaboration with nurses and maintain contact with family carers	Agree about collaboration and form of contact with GP Annual meeting of GPs and pharmacists to clarify current problems in prescription of medication and delivery Eliminate concerns and ambiguities about medication; inform and advise patients and doctors about proper use, possible side effects and interactions, storage and disposal Medication management Involved in interdisciplinary care of patients with polypharmacy
United Kingdom	Lead medication review Carry out medication reconciliation	Lead medication review Carry out medication reconciliation
Wales	No specific roles identified	No specific roles identified
Scotland	Combined knowledge and experience of physician, pharmacist, nurse and patient are required to ensure optimal treatment	Combined knowledge and experience of physician, pharmacist, nurse and patient are required to ensure optimal treatment
Australia	Identify clinical need Decide to refer patient for medication review Assess report and, in consultation with patient, prepare treatment plan	Identify clinical need Conduct comprehensive medication management reviews as part of their role in practice settings Initial and additional follow‐up Additional roles: patient‐directed roles, clinician‐directed roles, and system‐ or practice‐directed roles.

Abbreviation: GP, general practitioner.

In addition to the leading roles of the GP and the community pharmacist, several guidelines suggested an additional role for the (practice) nurse or pharmacy assistant in collecting patient information (The Netherlands, Japan), medication reconciliation (United Kingdom), assessing clinical need (Australia), but also in discussing the medication management plan and follow‐up (The Netherlands). In one guideline (The Netherlands), the involvement of specialists was recommended when additional information was required. One guideline (Mexico) targeted all medical professionals prescribing medication, including medical specialists and GPs. Only one guideline (Japan) mentioned that dentists play a role in medication management, as a team member for prescription review. Three guidelines (The Netherlands, United Kingdom, Australia) included specific recommendations about a multidisciplinary approach.

### 3.6 Patient Involvement

Seven of the eight guidelines included recommendations on patient involvement in medication management (Table 5). Of which six (The Netherlands, Germany, United Kingdom, Wales, Scotland, Australia) included recommendations to elicit the patient's experiences, views and preferences. Three guidelines (Germany, United Kingdom, Australia) recommended exploring the patient's desired level of involvement in the process, and one guideline (Wales) mentioned avoiding making assumptions about what matters to the patient. In six guidelines, shared decision‐making was mentioned as an underlying framework for involving the patient (Table 5). Two guidelines (United Kingdom, Scotland) recommended using patient decision aids. One guideline (Japan) recommended educating patients about their medication, mentioning patient‐centered medicine as the underlying framework.

**TABLE 5:** Recommendations and underlying frameworks for patient involvement in medication management per guideline (*n* = 7 out of 8 guidelines).

	Patient involvement	
Guideline	Recommendations	Underlying framework
The Netherlands	Talk with patient about actual drug use and drug‐related problems, concerns and expectations (e.g., pharmacotherapeutic anamnesis) Discussion of therapeutic goals with the patient Feedback of proposed changes to the patient and adjustment based on the patient's response	Not stated
Japan	Discontinuation of polypharmacy requires education to patients	Patient‐centered care
Germany	Ask patients about their treatment goals: improving quality of life, independence, function, prognosis, pain relief, improving symptoms, stress caused by medication The process of finding out patient preferences requires several steps: 1. recognizing situations for preference‐sensitive decision, 2. ensuring patients are adequately informed about the benefits and harms, 3. provide sufficient information, 4. find out desire of involvement in the decision‐making process	Shared decision‐making
United Kingdom	Offer patients the opportunity to be involved in making decisions about their medication. Find out what level of involvement the patient desires. Take into account the patient's: views and understandings about, and concerns, questions or problems with, the medication Find out about patient's values and preferences by discussing what is important to them when managing their conditions and their medication. Apply principles of evidence‐based medicine when discussing available treatment options In consultation about medication, offer patients opportunity to use patient decision aid (when one is available) to help make preference‐sensitive decision	Shared decision‐making
Wales	Find out what patient hopes treatment will achieve Listen, note non‐verbal cues and do not make assumptions about patient's preference for treatment Explain medical condition clearly and help patient make decisions based on likely benefits and risks rather than misconception Encourage and support patient to keep up‐to‐date list of medication Ask patient what they know and believe about their medication, including concerns Provide clear information Assess adherence At agreed intervals review patient's knowledge, understanding and concerns	Shared decision‐making
Scotland	Identify what matters to the patient. Identify aims and objectives of drug therapy. Explain key information and establish treatment objectives with the patient Find out if patient is willing and able to take drug therapy as intended. Does the patient understand the outcome? Adapt to patient preferences, agree and communicate the plan with patient Patients play vital role if provided with right information, tools and resources to make informed decisions about their medication	Shared decision‐making
Australia	Involve patients in decision‐making to the extent that they choose or are able to. This includes considering options, benefits and risks of medication In consultation discuss the patient's health‐related concerns, beliefs, attitudes and preferences In consultation obtain information from patient to inform comprehensive medication management review report Provide education and support to patient so they can make better‐informed choices	Shared decision‐making

### 3.7 Implementation

Three (The Netherlands, Germany, United Kingdom) guidelines included recommendations on how to implement their recommendations in clinical practice. The Dutch guideline contained an implementation plan at several levels, including a patient questionnaire to prepare patients for the medication review, agreements to be made between GPs and community pharmacists, and a format for the pharmacotherapeutic treatment plan. The German guideline recommended the implementation of a practice routine to identify patients who meet the criteria for a medication review. This routine could be supported by a pop‐up window in the patient's electronic medical record when a medication review is required. A summary version of the guideline with key recommendations was provided to support implementation. The United Kingdom guideline stated that better use of data and technology is needed to support implementation, and collaboration across health and social care sectors. For medicines reconciliation, a reference to an external guide for implementation is made [37].

### 3.8 Quality Appraisal

**FIGURE 2:**
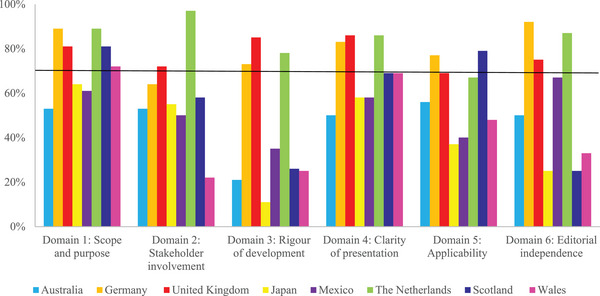
Scaled domain scores by AGREE‐II of all included guidelines (*n* = 8). The black line visualizes the lower limit for adequate domain scores. Scores above the line (≥ 70%) represent adequate quality of the corresponding domain.

Only three guidelines (The Netherlands, United Kingdom, Germany) scored above 70% for five out of six domains. The other five guidelines were below 70% for most or all domains. These guidelines (Australia, Japan, Mexico, Scotland, Wales) scored particularly low on rigor of development (11%–35%) and three of these guidelines (Japan, Scotland, Wales) also scored low on editorial independence (25%–33%) (Figure 2).

## 4 Discussion

Our scoping review provided an overview of medication management recommendations on polypharmacy from eight national guidelines [28, 30, 31, 32, 33, 34, 35, 36]. The most commonly recommended intervention was to conduct a structured, clinical medication review for a target population, including older patients or patients taking multiple medications. Recommendations on how to conduct the medication review varied, but the GP and the community pharmacist were recommended to play a crucial role. Other healthcare professionals to be involved in medication reviews, included (practice) nurses, medical specialists, pharmacy assistants and dentists. Six guidelines recommended patient involvement in medication management, most commonly through general statements on the importance of shared decision‐making. One guideline limited patient involvement to recommendations on patient education. Advice or guidance on the implementation of the recommendations was given in three guidelines. Applying the AGREE‐II criteria, three out of eight guidelines scored above 70% in five out of six domains.

Among the eight guidelines relevant to the clinical management of polypharmacy in primary care we identified were four new and two updated guidelines since the publication of a previous guideline review [10]. Similar to that review [10], our analysis showed that conducting a structured medication review is the most common strategy recommended in guidelines.

The description of the steps involved in conducting a medication review differ somewhat between guidelines. Few include all the steps described in a recent framework to optimize prescribing quality and minimize inappropriate medication in older people [38]. Some guidelines allowed for less extensive medication reviews without clear recommendations for whom or how to conduct these [31, 33]. In addition to medication reviews, previously recommended strategies for polypharmacy management include using explicit criteria or screening tools, such as the STOPP‐START or Beers criteria, comprehensive geriatric assessment and computerized decision support systems [9, 39]. Although such strategies were mentioned in some of the guidelines, our review showed that there appeared to be no universal approach for polypharmacy management.

It was recognized that medication management in older people with multimorbidity and polypharmacy is challenging and that medication reviews are not conducted in all patients who may need them [39]. A recent literature review identified several subgroups that could experience benefits from a medication review, including patients at risk (frail, recently hospitalized, or with multimorbidity) and patients using high‐risk medication [40]. Some guidelines did mention these subgroups, but there appeared to be no consensus on the target population for the conduct of medication reviews [40]. More research would be needed to develop valid selection criteria for the efficient conduct of medication reviews [41].

Most guidelines provided recommendations on patient involvement, focusing on eliciting patient preferences, concerns and treatment goals. Advice about incorporating patient preferences into decision‐making was observed particularly when the evidence supporting a recommendation was weak [42]. This might often be the case when making decisions about continuing or stopping medication in patients with polypharmacy. Although shared decision‐making was often mentioned as an underlying framework, only three guidelines made explicit recommendations to explore the extent to which patients wanted or could be involved in decision‐making.

Previously, it has been noted that guidelines for polypharmacy management mention different healthcare professionals [10]. Our review showed that there were no consistent recommendations regarding the role of various healthcare professionals in polypharmacy management in primary care. Several recent studies recommended a multidisciplinary approach, with clear roles and responsibilities, as an effective approach to medication management [43, 44, 45]. However, only three of eight guidelines in our review stated recommendations about a multidisciplinary approach. Although pharmacy‐led medication reviews could be successful [17], the level of implementation across Europe was limited [46]. Even though the research into the role of practice‐based pharmacist (or other types of primary care pharmacists) is emerging [47], none of the guidelines specified this in their recommendations. This might complicate the implementation of these recommendations. Subsequently, our review showed that guidelines did not provide much advice to support the implementation of recommendations.

Our review identified several knowledge gaps as well as similarities and differences between guidelines, which could be used for future development or improvement of guidelines for the management of polypharmacy in primary care. The most striking differences between guidelines were seen regarding the target population for performing a medication review, the definition of polypharmacy used in the guideline, the recommended composition of medication review, and the recommended additional healthcare professionals to be involved in managing polypharmacy.

Most guidelines recommended collaboration and consultation between the GP and the community pharmacist, but more guidance could be provided on task division and task delegation. It is notable that, even though evidence shows the effectiveness of a multidisciplinary approach, only a few guidelines include this in their recommendations. In addition, more evidence‐based methods for selecting patients are needed. There is also a need for more specific recommendations on involving patients in medication reviews through shared decision‐making. Extension of recommendations on implementing specific recommendations could help in clinical practice. To some extent, differences in guideline recommendations are expected, as the content depends on the context in which they are developed [48]. However, these differences may also result from consensus‐based instead of evidence based recommendations. Based on the results of the quality appraisal there is room for improvement in the development of guidelines, including stakeholder involvement, rigor of development, applicability and editorial independence.

We chose to conduct a scoping rather than a systematic review as it is a more appropriate method to identify key elements related to recommendations for medication management strategies provided in clinical practice guidelines [19]. Previous reviews focused on more specific research questions concerning, for instance, types of interventions [9], definitions of polypharmacy [49] or decision‐support systems used in the management of polypharmacy [50]. We focused on primary care, which has been identified as an important setting for polypharmacy management [9]. However, some of the guidelines included could apply to other settings, especially since not all of them were specifically developed for primary care settings. We used a broad and systematic search strategy including grey literature and accessing the team's network to find all relevant guidelines. Study selection, data extraction, and quality appraisal were performed independently and in duplicate. We used an iterative and systematic approach to identify key elements from the guidelines.

There are several limitations. First, we included guidelines limited by the language skills of the research team. We found only one guideline in another language, Swedish [51]. Although we included guidelines from a range of countries and in various languages, we did not identify any guidelines from low‐ and middle‐income countries (LMICs). Growing evidence suggests that overuse of medication is extensive in LMICs but evidence on solutions is still lacking [52]. In addition, our search was restricted to clinical practice guidelines. As a result, recommendations that were not published in guidelines have likely been excluded from our review. In the guidelines, recommendations were not always clearly specified and were presented with different wording in various parts of the guidelines. By duplicate data extraction, we expect to have included all relevant recommendations. However, there may be some subjectivity in the interpretation of the elements as described in the results. Finally, in some guidelines the lower quality appraisal scores were mostly due to insufficient reporting and may be an underestimation of the quality and the rigor of the actual development process. Other appraisal tools for clinical practice guidelines available, such as the GLIA‐tool or the ADAPTE‐tool, are more specific to selected dimensions of quality appraisal. AGREE‐II has been shown to be the most comprehensively validated quality appraisal tool [53].

In conclusion, medication review was the most recommended strategy to reduce inappropriate polypharmacy. The GP and the community pharmacist should play a prominent role, but there was no consensus on the division of tasks or on which population to address. The observed lack of agreement between guidelines and the low quality of the guideline development process offered opportunities to improve guidelines for polypharmacy in primary care further. In future guidelines, more explicit guidance would be needed on using a multidisciplinary approach with clear division of tasks between different healthcare professionals, and the involvement of patients in medication management through shared decision‐making. Additional research would be needed to support evidence‐based guidance on these topics. It would be recommended that LMICs be included in future research about polypharmacy.

## Conflicts of Interest

The authors declare no conflicts of interest.

## Supporting information



Supporting Information
