# Supplementary material for: Medication Management in Patients With Polypharmacy in Primary Care: A Scoping Review of Clinical Practice Guidelines
Source: J Evid Based Med. 2025 Mar 20;18(1):e70015. doi: 10.1111/jebm.70015 (PMC11923579; doi:10.1111/jebm.70015)
Supplement: Supplementary file 1 — Supporting Information [file JEBM-18-0-s001.docx]

**Supplementary Materials**

Supplementary Table 1. Inclusion and exclusion criteria

| **Inclusion criteria** | **Exclusion criteria** |
| --- | --- |
| Clinical practice guidelines | Other type of guidance documents:  Handbooks,  Positions statements,  Best practice reviews,  Guiding principles (documents that summarize existing guidelines) |
| Recommendations on medication management | Disease-, symptom- or drug-specific recommendations |
| Focus on population of adults with polypharmacy | (Sub)population specific guidelines:  Social care,  End of life care,  Cancer care,  Frailty |
| Applicable in primary care | Not applicable in primary care (e.g. trauma, surgery) |
| Aimed at healthcare professionals | Aimed at patients only |
| Language: English, Dutch, German, French, Spanish or Russian | All other languages |
| Publication date after the year 2000 | Publication date before the year 2000 |

Supplementary Material A. Search strategies

**Guidelines International Network:**

(polypharmacy) OR (medication management) OR (medication review)

**Turning Research into Practice:**

(polypharmacy OR polymedication OR multimedication) AND (medication management OR medicines management OR medication review OR medicines review OR medicines optimisation OR medication optimisation OR prescribing).

Filters: guideline, primary care.

**PubMed:**

((((((medication management) OR (medicines management)) OR (medication review)) OR (medicines review)) OR (medicines optimisation)) OR (medication optimisation)) OR (prescribing) AND ((polypharmacy) OR (polymedication)) OR (multimedication)

Filters: Practice Guideline, Dutch, English, French, German, Russian, Spanish, from 2000 – 2022.

Supplementary Material B. Data extraction framework

| **Characteristics** | |
| --- | --- |
| Publication | Author(s)/organization(s) (write text) |
|  | Year of publication (write number) |
|  | Country of origin (write text) |
|  | Title of the guideline (write text: translation [original]) |
| Context | Aim of the guideline (write text) |
|  | Definition of polypharmacy used in the guideline (write text) |
|  | Target (patient)population of the guideline (write text) |
|  | Target setting of the guideline (write text) |
| Development | Stakeholders/professional associations involved in development of the guideline (write text) |
| **Recommendations** | |
| Guideline | Country + year (write text) |
| Medication management | What are the different recommendations on medication management (including medication reviews)? (write text) |
|  | What intervention is recommended and how should it be performed? (write text) |
|  | When should the intervention be initiated in the process? And with whom? (write text) |
|  | Who should initiate the intervention? (write text) |
|  | What are the recommendations on follow-up on the intervention? (write text) |
|  | Notes (write text) |
| Healthcare professionals | Which healthcare professionals are recommended to be involved with medication management? (write text) |
|  | And what is their role? (write text) |
|  | Notes (write text) |
| Patient involvement | Are there any recommendations made on patient involvement in the process? (yes/no) |
|  | When should the patient be involved in the process? (write text) |
|  | Who should involve the patient in the process? (write text) |
|  | How should the patient be involved in the process? (write text) |
|  | What was the underlying framework for these recommendations? (write text) |
|  | Notes (write text) |
| Implementation | Are there any specific recommendations on implementation of the guideline in practice? (yes/no) |
|  | What are these recommendations? (write text) |
|  | Notes (write text) |
